# Supplementary material for: BET Bromodomain Degradation Disrupts Function but Not 3D Formation of RNA Pol2 Clusters
Source: Pharmaceuticals (Basel). 2023 Jan 29;16(2):199. doi: 10.3390/ph16020199 (PMC9966215; doi:10.3390/ph16020199)
Supplement: Supplementary file 1 [file pharmaceuticals-16-00199-s001.zip › pharmaceuticals-2065324-supplementary.pdf]

Supplemental figures for

BET bromodomain degradation disrupts function but not 3D formation of RNA Pol2 clusters

Diana H. Chin*[1], Issra Osman*[1], et al

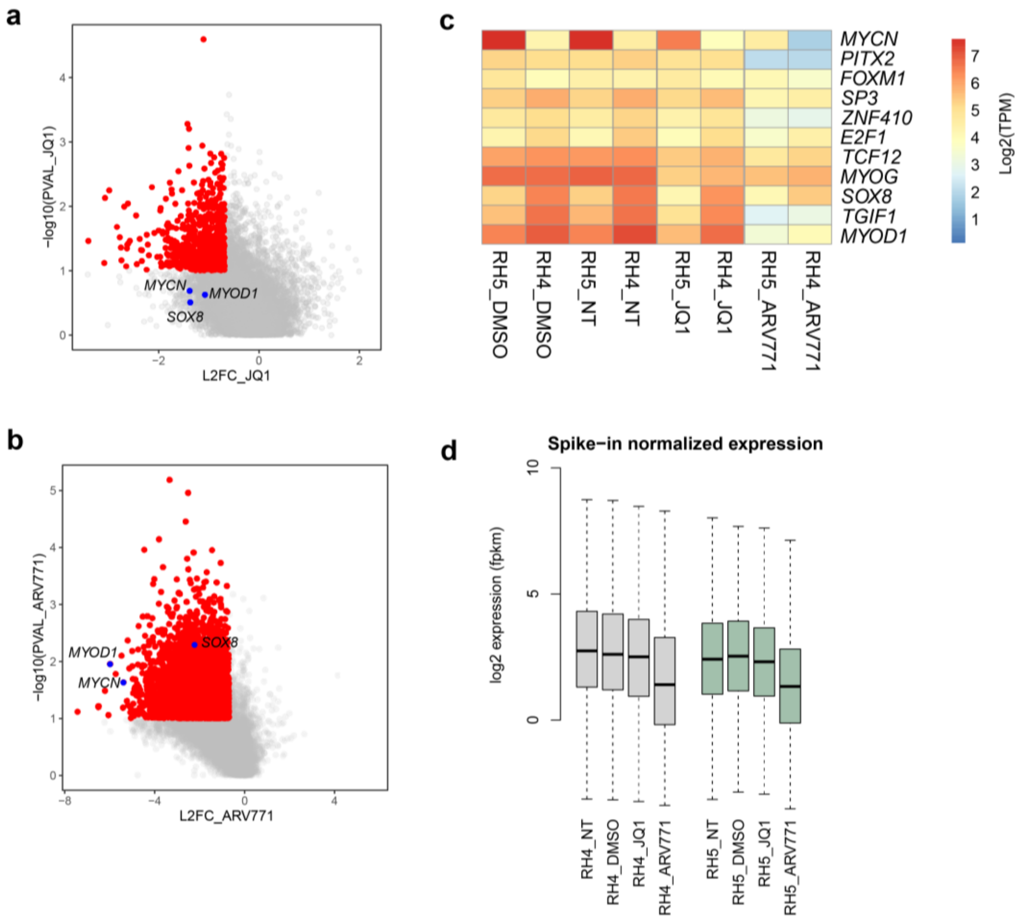

Supplemental Figure S1: Gene expression following loss of BET bromodomain proteins shows global decrease with degradation, but not inhibition.

a-b. Genes are plotted based on log2 fold change (L2FC) and the negative log10 of the p-value comparing TPM with JQ1 (a) or ARV-771 (b) treatment to TPM with DMSO treatment. Each gene is represented by one dot. Genes with L2FC less than -1 (fold-change < 0.5) and -log10(p-value) greater than 1 (p-val < 0.1) are shown in red. CRTFs MYOD1, MYCN, and SOX8 are highlighted.

c. Heatmap with log2 of total TPM for key genes in RH4 and RH5 treated with DMSO, JQ1, or ARV771. Red represents more transcripts, blue represents fewer. RH5 harbors amplified MYCN.

d. Boxplot with log2 of total FPKM for RH4 (gray) and RH5 (green) treated with DMSO, JQ1, or ARV771.

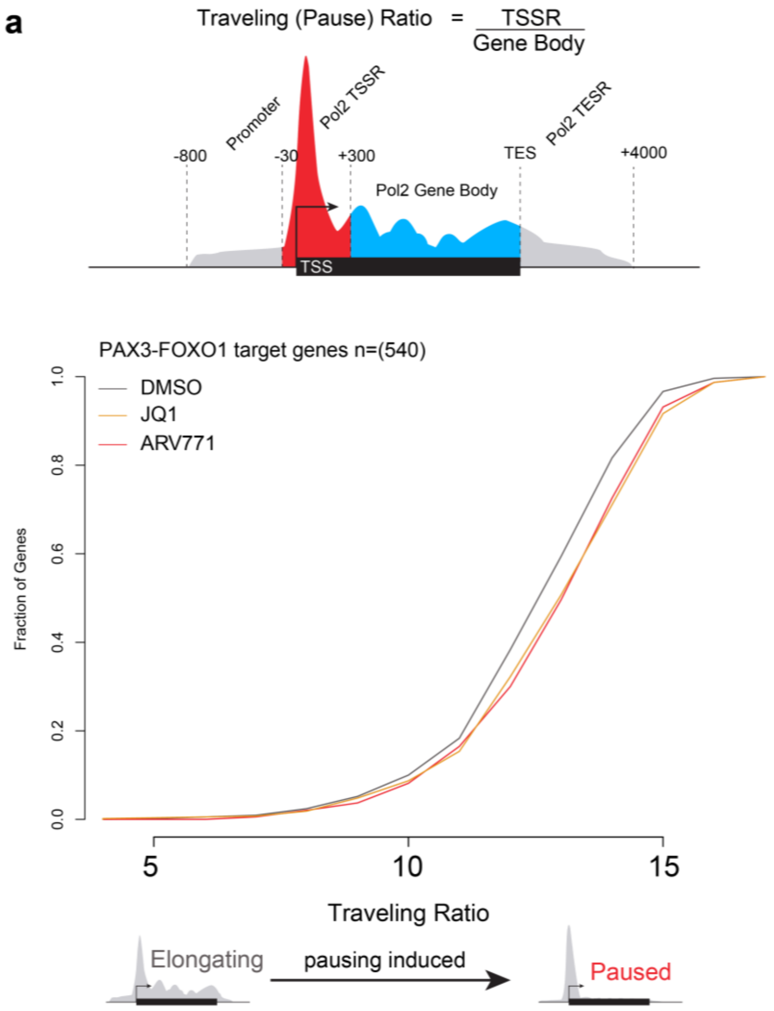

Supplemental Figure S2: Pol2 distribution within P3F targets upon the loss of BET bromodomain proteins.

a. Diagram of RNA Pol2 distribution as measured by the traveling ratio in a set of P3F target genes for RH4 cells treated with DMSO, JQ1, or ARV771. The traveling ratio is calculated by the ratio of reads at the transcriptional start site region (-30 to +300bp from the TSS) and the gene body (+300 from TSS to TES).

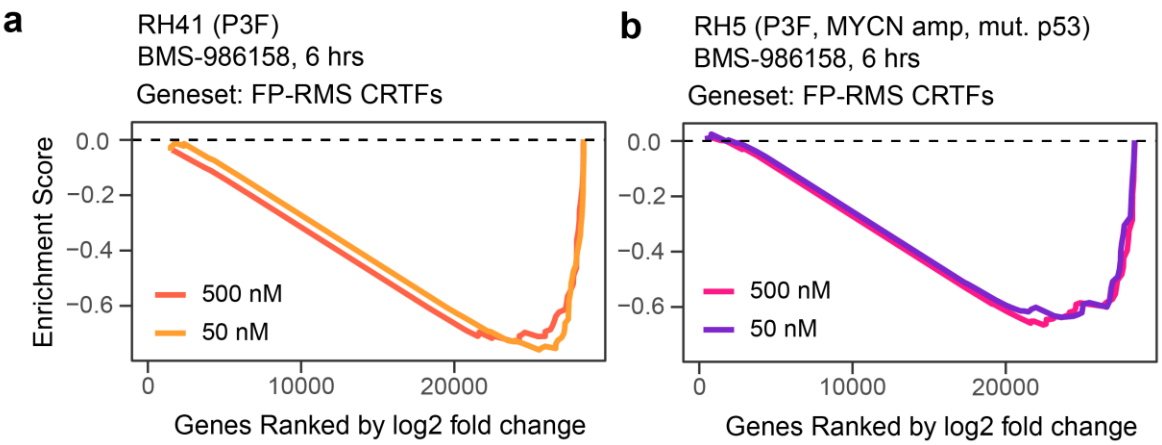

Supplemental Figure S3: BMS-986158 gene expression response in FP-RMS.
(a) Gene set enrichment analysis (GSEA) of RH41 and (b) RH5 cells treated shows strong downregulation of FP-RMS CRTFs following BMS-986158 treatment.
